# Bacterial evolution on demand

**DOI:** 10.7554/eLife.68070

**Published:** 2021-04-06

**Authors:** Pål J Johnsen, João A Gama, Klaus Harms

**Affiliations:** 1 Department of Pharmacy, Faculty of Health Sciences, The Arctic University of NorwayTromsøNorway

**Keywords:** *P. aeruginosa*, experimental evolution, antibiotic resistance, integron, demand, Other

## Abstract

Bacteria carry antibiotic resistant genes on movable sections of DNA that allow them to select the relevant genes on demand.

**Related research article** Souque C, Escudero JA, MacLean RC. 2021. Integron activity accelerates the evolution of antibiotic resistance. *eLife* **10**:e62474. doi: 10.7554/eLife.62474

Bacteria are the most abundant form of life and inhabit virtually every environment on Earth, from the soil to the human body. They display remarkable genetic flexibility and can respond rapidly to environmental changes. Bacteria have developed different strategies to increase their genetic diversity, including the use of mobile genetic elements, which can either move around the genome or be transferred to a different bacterium. These mobile genetic elements enable bacteria to acquire new genes that have already run the gauntlet of natural selection in other bacterial species – exemplified by the rapid emergence and global spread of antibiotic resistance.

However, producing resistance proteins in the absence of antibiotics, or acquiring resistance genes that are not yet needed, is costly and may reduce the viability of a bacterium. One way to counterbalance this burden is a genetic element known as the integron. Integrons are genetic platforms that can capture and shuffle genes, thus providing instant adaptive benefits in fluctuating environments (Stokes and Hall, 1989).

Integrons are not mobile in their own right, but they are often embedded within mobile genetic elements that can facilitate their transfer. They contain an array of antibiotic resistant genes known as gene cassettes. The ones located at the start of the integron produce more proteins than the ones closer to the end (Collis and Hall, 1995). During stress situations – such as exposure to antibiotics – an enzyme called integrase is produced, allowing the microbes to shuffle the order of the cassettes in the integron (Guerin et al., 2009). Consequently, gene cassettes encoding the appropriate adaptive response to an antibiotic will be moved closer to the start, where expression levels are higher (Figure 1). It has been proposed that this mechanism allows bacteria to adapt to fluctuating environmental conditions ‘on demand’, but this hypothesis has never been experimentally tested (Escudero et al., 2015).

**Figure 1.**
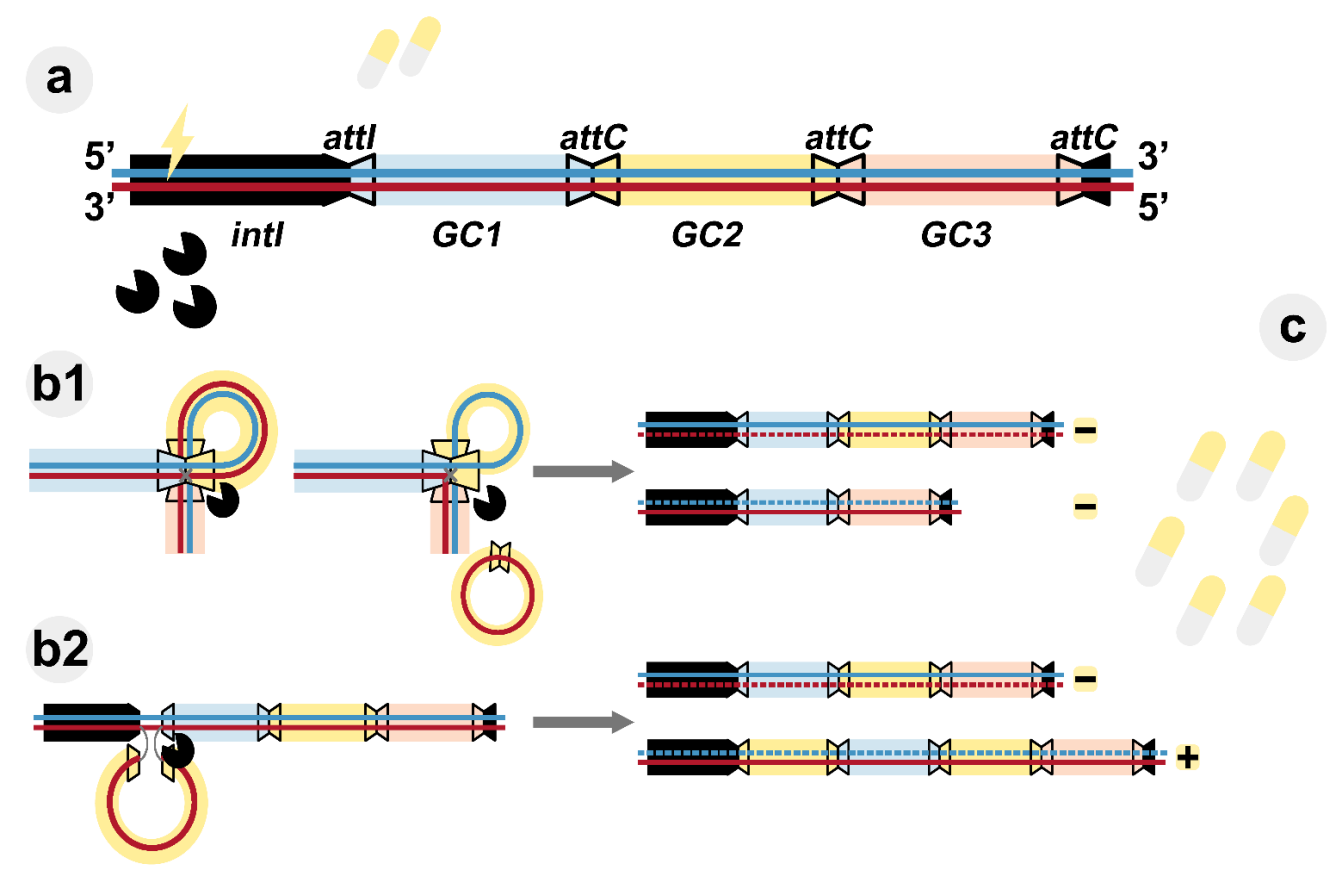
Evolutionary dynamics of integrons. (**A**) Class-1 integrons consist of a 5'-conserved region (black section) and an integrase (*intI*) that can capture gene cassettes (GC, colored rectangles) and insert them at the *attI* gene site. They are the most wide-spread class of these genetic elements and contain arrays ranging from one to five gene cassettes. The gene cassettes are separated by *attC* sites (trapezoids with black borders). Exposure to low doses of antibiotic (yellow tablets) activates the integrase (pacman symbols). (**B**) The integrase can rearrange the gene cassettes by either cutting them at *attC* sites (**b1**), or by inserting previously excised or free gene cassettes at the *attI* site (**b2**). The integrase acts by breaking and rejoining the bottom DNA strand (red). After DNA replication (gray arrows), daughter integrons are parental (derived from the blue top strand) or carry a changed gene cassette array (derived from the bottom strand). (**C**) Increased antibiotic concentrations exert selective pressure on the diversity of the generated integron. Those carrying the appropriate gene cassette (yellow rectangle) in the first position are favored (+) due to higher expression of resistance, while other gene cassette arrays are counter-selected (–).

Now, in eLife, Célia Souque, José A. Escudero and Craig MacLean of the University of Oxford and the Universidade Complutense de Madrid report new insights into how bacteria evolve on demand when exposed to antibiotic treatment (Souque et al., 2021). Souque et al. used two strains of the bacterium *Pseudomonas aeruginosa*, differing only by the presence or absence of a functional integrase. Both strains contained an integron with three gene cassettes encoding resistance to different antibiotics, with the relevant resistant gene in the last position.

The researchers used an experimental evolution protocol known as the evolutionary ramp design. In this set up, bacteria are challenged with daily doublings of antibiotic concentrations, which force the bacterial populations to rapidly increase their antibiotic resistance levels or face extinction. After 13 concentration ramps with the antibiotic gentamicin, significantly more populations containing a functional integrase survived than their integrase-negative counterparts, which largely went extinct.

The ability to move the required gentamicin-resistance gene cassette forward in the integron to increase resistance levels may at first resemble adaptive evolution with a neo-Lamarckian flavor. However, examining the genetic sequence of the earlier evolved strains (that arose at the start of the ramp experiment) revealed a range of cassette arrays, with the three gene cassettes in various positions. This suggests that integrase activity generates random genetic diversity upon which natural selection could act.

In fact, structural variation was found in half of the evolved populations early in the evolutionary ramp experiments. But at the highest antibiotic concentrations, most of them contained a single variant i.e., they all contained the same cassette array. However, the evolved control populations that were either not exposed to antibiotics or only subjected to low concentrations, did not show any structural variation in the cassette arrays. Taken together, this provides compelling evidence favoring the ‘evolution on demand hypothesis’.

This study represents an excellent example of how hypotheses concerning the evolution and maintenance of mobile genetic elements can be tested using combined approaches of experimental evolution, population biology and genetics. To that end, Souque et al. also propose new mechanistic insights into the recombination dynamics within integron cassette arrays. They found integrons with gentamicin-resistance cassettes both in first and last position, suggesting a ‘copy and paste’ mechanism of the integrase, rather than a ‘cut and paste’ one.

Duplicated gene cassettes indeed exist in integron-containing bacterial isolates from environmental and clinical samples, but they are likely rarer than the proposed preferential ‘copy and paste’ mechanism. This could be due to the suggested short-read sequencing bias. Another possibility could be that gene cassettes are excised from other integrons within the same cell, and then integrated into the first position.

In our opinion, whether integrase activity mechanistically leads to ‘copy and paste’ versus ‘cut and paste’ could only be resolved using experimental assays with a single integron, but we acknowledge that this is difficult to obtain. Even when integrons are located on the chromosome, multiple copies will be present just before cell division.

Finally, Souque et al. also addressed the question why the integrase enzyme is costly (Starikova et al., 2012; Lacotte et al., 2017). This has previously been linked to off-target integrase activity resulting in deleterious genetic rearrangements (Harms et al., 2013). The fact that most integrase-containing populations in this study rarely undergo genetic shuffeling suggests that off-target integrase activity is rare and that other not-mutually exclusive mechanisms could explain the costly nature of active integrases.

Understanding the evolutionary dynamics of these fascinating integrons and the benefits they provide to bacteria may help us on the quest of finding new treatment strategies that limit the evolution of antibiotic resistance.
